# Can a virtual microbiology simulation be as effective as the traditional Wetlab for pharmacy student education?

**DOI:** 10.1186/s12909-021-03000-3

**Published:** 2021-11-17

**Authors:** L. Baumann-Birkbeck, S. Anoopkumar-Dukie, S. A. Khan, M. J. Cheesman, M. O’Donoghue, G. D. Grant

**Affiliations:** 1 School of Pharmacy and Medical Sciences, Griffith University, 1 Parklands Dr, Southport, QLD 4215 Australia; 2 Menzies Health Institute, G40 Griffith Health Centre, Level 8.86, Griffith University, 1 Parklands Dr, Southport, QLD 4215 Australia; 3 Quality Use of Medicines Network, Griffith University, 1 Parklands Dr, Southport, QLD 4215 Australia; 4 Mater Research Institute, The University of Queensland, Raymond Terrace, Level 3 Aubigny Place, South Brisbane, QLD 4101 Australia; 5 Squina International Centre for Infection Control, School of Nursing, The Hong Kong Polytechnic University, Room FJ502, Hung Hom, Kowloon, Hong Kong, Special Administrative Region of China

**Keywords:** Simulation, Pharmacy, Practice, Education, Clinical, Microbiology

## Abstract

**Background:**

Pharmacy practice education requires the development of proficiencies and an understanding of clinical microbiology. Learning in this area could be delivered using practical laboratory exercises, or potentially, simulation-based education. Simulation has previously successfully enhanced learning in health professional education. The current global climate due to COVID-19 has further highlighted the important role of technology-enhanced learning in delivering outcomes that meet the requisite learning objectives of a course. The aim of the present study was to compare the impact of a commercially available virtual microbiology simulation (VUMIE™) with a traditional wet laboratory (wetlab) on learner knowledge, skills and confidence in a second-year integrated pharmacotherapeutics course for Bachelor of Pharmacy students.

**Methods:**

A randomised, crossover study was employed to determine whether the simulation intervention (VUMIE™) improves learning outcomes (knowledge, skills and confidence) of pharmacy students, when compared to a traditional wetlab intervention. Each student completed three 1–2 h length sessions, for both the wetlab and VUMIE™ interventions (6 sessions total). Data was collected using surveys deployed at baseline (pre-interventions), post-intervention 1 or 2 (VUMIE™ or wetlab) and endpoint (post-interventions 1 and 2). Statistical analysis was conducted using SPSS Statistics 25 and Instat™ software.

**Results:**

Response rates were approximately 50% at initial survey and approximately 25% at endpoint survey. VUMIE™ produced higher post-intervention knowledge scores for the multiple-choice questions compared to the wetlab, however, the highest score was achieved at endpoint. Both interventions produced statistically significant differences for mean scores compared to baseline (pre-VUMIE™ and wetlab) across the domains of knowledge, skills and confidence. VUMIE™ produced higher post-intervention mean scores for knowledge, skills and confidence compared to post-intervention mean scores for the wetlab, however there was no statistical significance between the mean score for the two interventions, thus the VUMIE™ activity produced learning outcomes comparable to the wetlab activity.

**Conclusion:**

These findings suggest VUMIE™ provides similar effects on students’ knowledge, skills, and confidence as a wetlab. The simulation’s implementation was not cost-prohibitive, provided students with a physically and psychologically safe learning environment, and the benefit of being able to repeat activities, supporting deliberate practice.

## Background

Pharmacy practice education requires the development of discipline-specific knowledge, skills, and capabilities [1]. Replacement or adjunct use of simulations or technology-enhanced learning activities is becoming more common in clinical programs to reduce laboratory associated costs, relieve placement sites and facility burden, and to provide flexible, repeatable delivery options for students to acquire mastery of the content, skills, and capabilities [2, 3]. The development of clinical microbiology laboratory skills is recognised as a ‘speciality area’, not routinely required for everyday pharmacy practice [4, 5]. Pharmacy practice mostly involves the clinical aspect of microbiology, core knowledge of the skills and practice underpinning antibiotic selection and use should be demonstrated by graduates. Although practising pharmacists should understand these core elements to deliver quality use of medicines, manual skillsets involved in such specialty areas, like microbiology, may not be required [6]. To create an understanding, the information regarding practical areas (not taken on in a typical pharmacy practice role) could be achieved using practical laboratory exercises, or potentially, simulation-based digital education modalities. In addition, the current climate surrounding the COVID-19 pandemic, and the need for replacement of face to-face learning activities with flexible computer-based platforms, requires education facilitators to teach students through on-line tools [7]. Educators have been afforded very little time to prepare for such online learning, so an awareness of available software programs is beneficial when facilitating delivery of learning that is not able to be conducted face-to-face [8, 9].

Previously the cost of simulation training was high, however they have proven to be a very flexible and durable form of clinical education and training [10, 11]. Simulation-based education offers advantages including saving on consumables and promoting learning flexibility. Simulations can expand student opportunities to gain clinical skills, despite the challenges of finding clinical placement/practice sites, which has resulted from large student numbers as well as ethical and indemnity issues that arise when student actions may have negative consequences for real patients [3, 12, 13]. Technology-enhanced clinical education can also provide greater efficiency and opportunities for diligence, compared to the limited opportunities afforded by clinical and practical experiences, and can be flexibly scheduled and repeated as necessary to allow learning consolidation through deliberate practice [14, 15].

Feasibility and acceptability of implementing virtual simulation for education in health fields has been noted as a significant issue [16, 17]. Researchers have noted that where simulation has been considered a replacement for face-to-face learning, the modality needs to provide similar learning outcomes for students compared to traditional or existing methods [18, 19]. Recent literature focuses primarily on these two issues, as researchers and academics seek to fully appreciate where simulation education should apply, not just can apply.

The aim of this study was to compare the impact of a commercially available virtual microbiology simulation (VUMIE™) with a traditional wetlab on learner knowledge, skills and confidence in a second-year integrated therapeutics course for Bachelor of Pharmacy students. Learning activities focused on a number of core clinical microbiology competency areas, including Gram staining, selection and use of media and biochemical tests, and susceptibility testing [20]. This research is timely, given the urgent need for education to be delivered in digital formats due to SARS-CoV-2.

## Methods

The VUMIE™ software was incorporated into a second-year integrated pharmacotherapeutics course in the Bachelor of Pharmacy degree, during the years 2016 to 2019. The data presented in this study is a collation of multiple cohort years who completed the course. VUMIE is an interactive digital microbiology application which simulates workflow in a microbiology laboratory using visually accurate workspace, equipment, and consumables. Ethical clearance was granted by the relevant human research ethics committee (HREC 2016/231). An experimental study (randomised, crossover) was employed to determine whether the simulation intervention (clinical skills training in a virtual environment with VUMIE™) improves knowledge, skills and confidence of pharmacy students, when compared to a traditional wetlab experience. Metrics were assessed by both self-reported measures and external assessment of knowledge. Students were allocated a license for the VUMIE™ software. VUMIE™ is delivered on a computer and requires an internet connection. Participation in the activity was compulsory, although completion of the surveys was voluntary. Each student completed three sessions of each activity (VUMIE™ and traditional wetlab). Sessions were 1–2 h in length, for both the wetlab and VUMIE™ intervention. The wetlab activities involved the identification of microbial organisms, including Gram staining, plating and growth of organisms, and testing susceptibility of organisms to antibacterial treatments, to inform clinical decision-making in patient cases. The VUMIE™ sessions involved activities aligned and comparable to wetlabs, however these were simulated with the virtual laboratory software, in a workshop classroom. Due to their interrelated nature, all activities were covered in each session of both interventions.

Students were randomly allocated into two groups using their student number, by a course administrator. The first group undertook traditional (wetlab) laboratory activities (three sessions completed over three weeks). The second group undertook a similar virtual laboratory activity using VUMIE™, completed over those same three weeks. Both groups then swapped over and completed the other respective activity over the following three-week period.

**Fig. 1:**
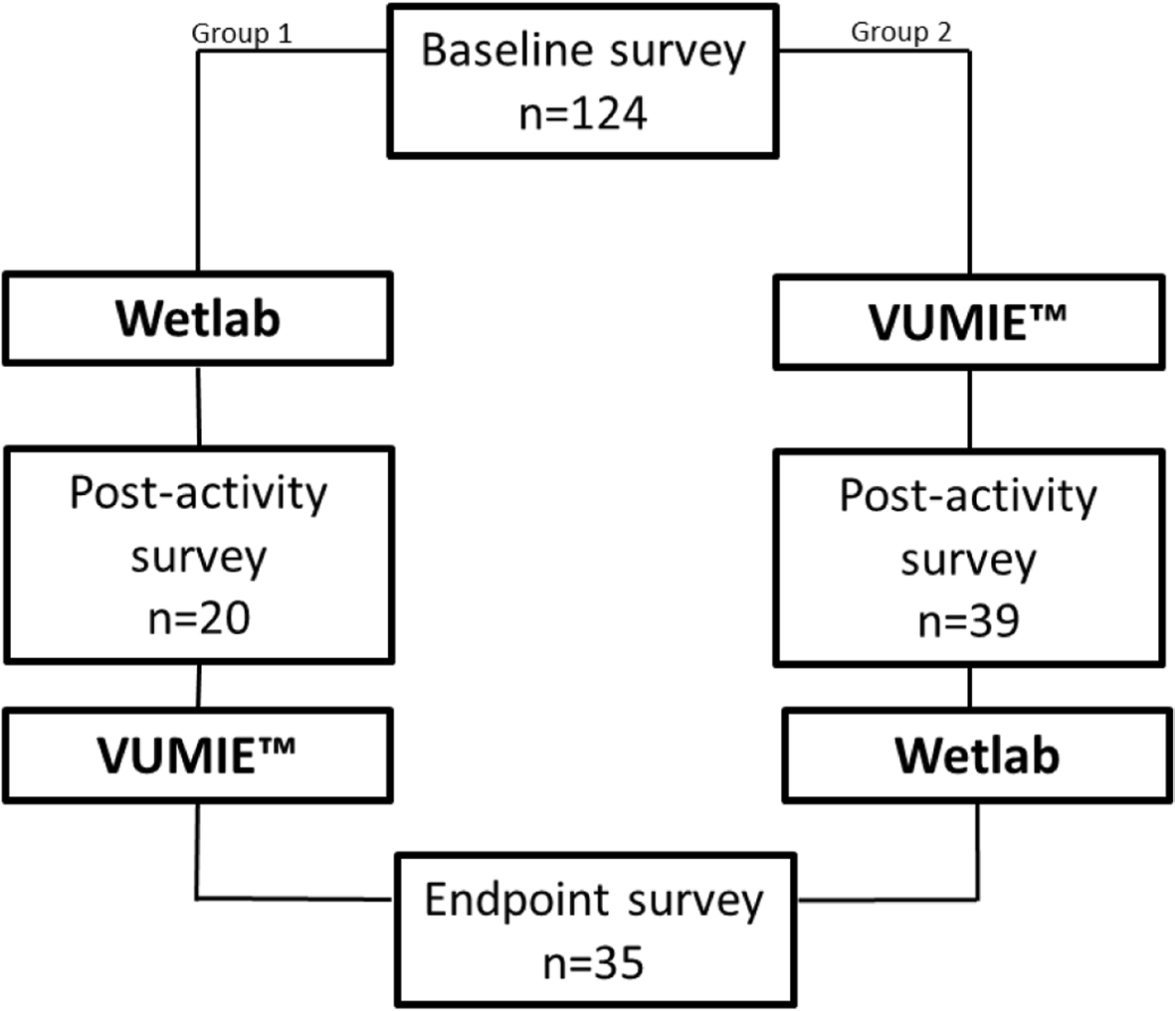
Flowchart of investigation and surveys completed

Students who consented to data collection were invited to complete a baseline survey, a survey following the completion of their first activity (wetlab or VUMIE™) and an endpoint survey, after completing both the wetlab and VUMIE™ (Fig. 1). The surveys were anonymous and coded to protect students’ identity. Three students’ responses were removed from the results as they were unable to participate in the wetlabs and completed the virtual activities only. The baseline survey included technology acceptance and detailed demographic data including grade point average (GPA), gender and prior laboratory/microbiology experience. Each survey required students to report a score on a 5-point Likert scale that they believed corresponded to their level of agreement or disagreement with a statement regarding their knowledge, skills and confidence in a number of topics relating to clinical microbiology. These topics included Gram staining, growth media, biochemical tests and susceptibility testing. The Likert scale included the following points; 1 = strongly disagree, 2 = disagree, 3 = neutral, 4 = agree and 5 = strongly agree. All questions on the post-intervention surveys were worded in the same way and directly comparable to the baseline survey. Examples of the survey items include, “I have the appropriate knowledge to perform a Gram stain”, “I have the appropriate skills to perform a Gram stain”, “I have the appropriate confidence to perform a Gram stain”. The surveys also contained four identical multiple-choice questions which examined knowledge of clinical microbiology which related directly to course content. Survey construction was based on the revised Bloom’s taxonomy and the Quality and Safety Education for Nurses (QSEN) model of competencies survey, which has informed a number of similar instruments [20, 21]. Comparable surveys were also piloted in prior studies on technology-enhanced simulation in pharmacy practice education, which provided further justification. Data was collected using Jotform. The items for knowledge, skills and confidence were tested for reliability using Cronbach’s alpha analysis on SPSS 25. The 16-item knowledge scale had good reliability with a Cronbach’s alpha of 0.971 (baseline), 0.964 (post-wetlab), 0.976 (post-VUMIE™) and 0.974 (endpoint). The 12-item skills scale also had a good reliability with a Cronbach’s alpha of 0.973 (baseline), 0.943 (post-wetlab), 0.972 (post-VUMIE™), and 0.966 (endpoint). The 12-item confidence scale demonstrated Cronbach’s alpha of 0.983 (baseline), 0.976 (post-wetlab), 0.970 (post-VUMIE™) and 0.966 (endpoint).

A total of 124 students consented to their data being collected and analysed for this study (84% participation). These students all completed the baseline questionnaire. Thirty-nine students completed the post-VUMIE™ survey and 20 students completed the post-wetlab activity after 3 weeks of the respective activity (response rate approximately 50%). Thirty-five students completed the endpoint survey after the total 6 weeks of the two activities, giving the study suitable power (80% power to detect a difference between means of 0.52 with a significance level (alpha) of 0.05 (two-tailed)). Learning outcome data were analysed using Instat™ software and statistical analyses of self-reported scores was conducted using SPSS Statistics 25. Cronbach’s alpha was employed to determine reliability for the survey items. Statistical comparisons between groups were performed by Mann Whitney test. Baseline outcomes were compared to post-wetlab, post-VUMIE™ and endpoint scores. Post wet-lab and post-VUMIE™ scores were also compared. Endpoint scores were compared to both post-wetlab and post- VUMIE™ scores.

## Results

The data is a collation of multiple cohort years who completed the course. Each cohort had approximately 40 students and therefore several cohorts’ data was collected to achieve adequate study power. Students who completed the surveys were on average, female, under 25 years of age, with a GPA between 4 and 6 (7 being highest, below 4 being a fail). The demographics of the participants was representative of the entire population of students that had completed the course during the study. Approximately 92% of students had completed a previous course which included aspects of microbiology, as part of their degree at university and they had spent an average time of 5–10 h in a laboratory (Table 1).

**Table 1:** Overview of participant demographics

Variable	Values	Number	Percent
Gender	Male	41	33
	Female	83	67
Age	< 25 years	103	83
	≥25 years	21	17
GPA	< 4.0	8	6
	4.0–6.0	97	79
	> 6.0	19	15
Previous microbiology course experience	Yes	114	92
	No	10	8
Hours spent in a laboratory	< 5 h	27	22
	5–10 h	48	39
	11–20 h	35	28
	> 20 h	14	11

### Knowledge, skills and confidence self-reported scores

Tables 2, 3 and 4 below show the data for student responses to the surveys, which were deployed at baseline, post-VUMIE™ or post-wetlab (depending on the activity assigned), and at the endpoint. Individual item overall scores (Gram stain, media, biochemical tests and susceptibility) for knowledge, skills and confidence were compared. The overall item (indicated in bold) required students to respond based on their ‘overall’ knowledge regarding the given topic. For example, a student’s response to ‘Gram stain – overall’ encompassed their self-reported learning outcome for the Gram stain process, performing a Gram stain and interpreting a Gram stain, considered holistically. For this reason, the overall scores for each main item (Gram stain, media, biochemical tests and susceptibility) were compared statistically, rather than separate responses (e.g. Gram stain process, interpreting a Gram stain etc.).

**Table 2:** Self-reported scores for the knowledge learning domain

KNOWLEDGE Learning outcome	Baseline *N* = 124 Mean (SD)	Post-wetlab *N* = 20 Mean (SD)	Post-VUMIE™ *N* = 39 Mean (SD)	Endpoint *N* = 35 Mean (SD)
Gram stain – Process	3.6 (0.99)	4.2 (0.77)	4.0 (0.99)	4.5 (0.56)
Gram stain – Perform	3.5 (1.03)	4.0 (0.83)	3.8 (1.03)	4.4 (0.60)
Gram stain – Interpret	3.5 (1.00)	4.2 (0.79)	4.4 (0.79)	4.5 (0.56)
**Gram stain – Overall**	**3.4 (1.04)**	4.0 (0.83)*	4.0 (0.92)**	4.4 (0.61)**** ^**#∆**^
Media – Types	3.2 (0.96)	3.9 (0.71)	4.1 (0.76)	4.3 (0.63)
Media – Choice	3.1 (0.98)	3.7 (0.73)	3.9 (0.81)	4.1 (0.87)
Media - Interpret	3.1 (0.95)	3.8 (0.63)	4.1 (0.77)	4.3 (0.66)
**Media – Overall**	**3.2 (0.97)**	3.7 (0.73)*	4.0 (0.79)****	4.2 (0.65)**** ^**#**^
Biochemical Tests – Type	3.1 (0.89)	3.8 (0.89)	4.1 (0.74)	4.3 (0.53)
Biochemical Tests – Choice	3.0 (0.91)	3.7 (1.04)	4.0 (0.78)	4.2 (0.63)
Biochemical Tests -Interpret	3.0 (0.89)	3.6 (0.99)	4.1 (0.72)	4.3 (0.57)
**Biochemical Tests – Overall**	**3.0 (0.92)**	3.7 (0.93)**	4.1 (0.76)****	4.2 (0.60)**** ^**#**^
Susceptibility – Determine	3.0 (1.02)	3.9 (0.72)	4.1 (0.81)	4.4 (0.61)
Susceptibility – Perform	2.9 (1.00)	3.9 (0.72)	4.1 (0.76)	4.3 (0.63)
Susceptibility – Interpret	3.0 (1.00)	3.9 (0.67)	4.0 (0.78)	4.4 (0.60)
**Susceptibility – Overall**	**3.0 (1.01)**	3.9 (0.72)****	4.1 (0.77)****	4.3 (0.63)**** ^**#**^

* *= p* < 0.05 (compared to baseline)

** = *p* < 0.01 (compared to baseline)

**** = *p* < 0.0001 (compared to baseline)

^#^ = *p* < 0.05 (compared to post-wetlab)

∆ = *p* < 0.05 (compared to post-VUMIE™)

**Table 3:** Self-reported scores for the skills learning domain

SKILLS Learning outcome	Baseline N = 124 Mean (SD)	Post-wetlab N = 20 Mean (SD)	Post-VUMIE™ N = 39 Mean (SD)	Endpoint N = 35 Mean (SD)
Gram stain – Perform	3.6 (0.95)	3.9 (0.72)	3.7 (1.02)	4.4 (0.36)
Gram stain – Interpret	3.6 (0.96)	4.1 (0.51)	4.3 (0.80)	4.6 (0.25)
**Gram stain – Overall**	**3.5 (0.97)**	**3.9 (0.64)**	3.9 (0.89)*	4.5 (0.26)**** ^**###∆∆**^
Media – Choice	3.1 (0.89)	3.7 (0.92)	4.0 (0.77)	4.1 (0.49)
Media – Interpret	3.2 (0.91)	3.9 (0.64)	4.0 (0.81)	4.2 (0.49)
**Media – Overall**	**3.1 (0.91)**	3.7 (0.88)**	4.0 (0.81)****	4.2 (0.55)**** ^**#**^
Biochemical Tests – Perform	3.2 (0.91)	3.7 (0.80)	4.0 (0.83)	4.2 (0.41)
Biochemical Tests -Interpret	3.2 (0.91)	3.7 (0.73)	4.0 (0.79)	4.3 (0.37)
**Biochemical Tests - Overall**	**3.2 (0.90)**	3.7 (0.73)**	4.0 (0.79)****	4.2 (0.42)**** ^**##**^
Susceptibility – Perform	3.0 (0.90)	3.8 (0.69)	4.0 (0.78)	4.4 (0.42)
Susceptibility – Interpret	3.0 (0.93)	3.8 (0.63)	4.1 (0.75)	4.4 (0.48)
**Susceptibility – Overall**	**3.0 (0.89)**	3.8 (0.63)****	4.0 (0.76)****	4.4 (0.48)**** ^**##∆**^

* = *p* < 0.05 (compared to baseline)

** = *p* < 0.01 (compared to baseline)

**** = *p* < 0.0001 (compared to baseline)

^#^ = *p* < 0.05 (compared to post-wetlab)

^# #^ = *p* < 0.01 (compared to post-wetlab)

∆ = *p* < 0.05 (compared to post-VUMIE™)

∆∆ = *p* < 0.01 (compared to post-VUMIE™)

**Table 4:** Self-reported scores for the confidence learning domain

CONFIDENCE Learning outcome	Baseline N = 124 Mean (SD)	Post-wetlab N = 20 Mean (SD)	Post-VUMIE™ *N* = 39 Mean (SD)	Endpoint *N* = 35 Mean (SD)
Gram stain – Perform	3.4 (0.98)	3.7 (1.00)	3.7 (1.08)	4.3 (0.37)
Gram stain – Interpret	3.4 (0.98)	3.9 (0.85)	4.2 (0.80)	4.5 (0.26)
**Gram stain – Overall**	**3.4 (0.97)**	3.8 (0.83)*	3.9 (0.85)**	4.4 (0.37)**** ^**##∆∆**^
Media – Choice	3.0 (0.91)	3.6 (0.94)	3.9 (0.79)	4.1 (0.48)
Media – Interpret	3.2 (0.95)	3.8 (0.83)	4.1 (0.79)	4.2 (0.48)
**Media – Overall**	**3.1 (0.91)**	3.7 (0.86) **	3.9 (0.79)****	4.1 (0.48)****
Biochemical Tests – Perform	3.1 (0.95)	3.7 (0.86)	3.9 (0.82)	4.3 (0.37)
Biochemical Tests -Interpret	3.1 (0.94)	3.7 (0.86)	4.0 (0.75)	4.3 (0.38)
**Biochemical Tests – Overall**	**3.1 (0.95)**	3.7 (0.86)**	4.0 (0.82)****	4.2 (0.42)**** ^**#**^
Susceptibility – Perform	3.1 (0.96)	3.9 (0.59)	4.0 (0.78)	4.3 (0.46)
Susceptibility – Interpret	3.1 (0.96)	3.9 (0.59)	4.1 (0.76)	4.4 (0.36)
**Susceptibility – Overall**	**3.1 (0.96)**	3.9 (0.59)***	3.9 (0.76)****	4.3 (0.46)**** ^**#∆**^

* = *p* < 0.05 (compared to baseline)

** = *p* < 0.01 (compared to baseline)

*** = *p* < 0.001 (compared to baseline)

**** = *p* < 0.0001 (compared to baseline)

^#^ = *p* < 0.05 (compared to post-wetlab)

^# #^ = *p* < 0.01 (compared to post-wetlab)

∆ = *p* < 0.05 (compared to post-VUMIE™)

∆∆ = *p* < 0.01 (compared to post-VUMIE™)

### Technology acceptance

To establish students’ attitudes toward technology, prior to use of VUMIE™, technology acceptance was surveyed at baseline. Student responses (*n* = 124) regarding technology acceptance were overwhelmingly positive, with over 90% (113) of respondents either willing or very willing to use technology ordinarily. Similarly, over 85% (107) of respondents reported that they felt having a virtual microbiology training tool available to them would be somewhat or very useful. The baseline survey results also reported that approximately 46% (57) of students reported feeling that a virtual microbiology program would be either somewhat useful or very useful *instead* of a practical microbiology laboratory.

### Multiple-choice knowledge questions results

Four identical multiple-choice questions were included in each survey. VUMIE™ produced higher post-intervention scores for the questions compared to the wetlab. Lowest scores were achieved at baseline and highest scores were achieved at endpoint.

## Discussion

### Learning outcomes

**Fig. 2:**
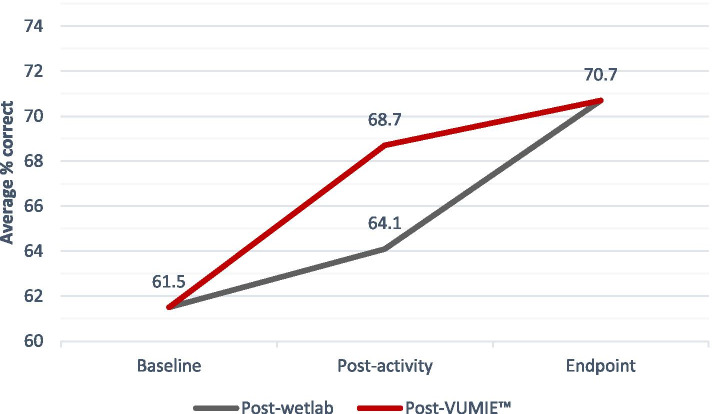
Average percentage correct answers for multiple-choice knowledge questions

Both interventions produced statistically significant differences in mean scores compared to baseline across the domains of knowledge, skills and confidence. As seen in Tables 2, 3 and 4, VUMIE™ produced higher post-intervention mean scores for knowledge, skills and confidence compared to post-intervention mean scores for the wetlab, however there was no statistical significance between the mean score for the two interventions. This suggests that the VUMIE™ activity produces learning outcomes that are comparable to the wetlab activity. The results of the multiple-choice knowledge questions also reflected that VUMIE™ produced higher post-intervention scores compared to the wetlab, however, the highest score was achieved at endpoint, as seen in Fig. 2. Additionally, statistically significant differences were also recorded for endpoint compared to post-wetlab for knowledge, skills and confidence, which suggests that completing the VUMIE™ activity in addition to the wetlab made a positive impact on student learning outcomes. Therefore, completion of both interventions is likely to be more beneficial for student learning than either activity alone. Of the individual items assessed in the surveys, the largest mean score was reported for Gram staining interpretation, again across all three learning domains. These findings are consistent with several other studies which demonstrate that virtual simulation can produce comparable learning outcomes compared to traditional teaching methods [15, 17, 22, 23]. The current COVID-19 pandemic has meant that some students’ progression in their degree has been stalled, due to the inability to complete practical laboratory components. A simulation, such as VUMIE™, which produces comparable results to traditional education modes, may allow some health programs to deliver teaching which previously required a traditional laboratory, thereby allowing students to complete pre-requisite modules that may otherwise have been postponed. Several studies have also reported the usefulness of simulation for education during the pandemic, and highlighted the benefit of globally collaborative efforts to continue to provide educational solutions like simulation, so that pharmacy students can still meet required learning outcomes, even when traditional learning environments are not feasible [24, 25].

Use of a virtual simulation also provides benefits for students who can repeat processes and skills that in a traditional wetlab they may only be able to practice once, due to time, cost, supervision and consumables availability. According to this study, the wetlab did not produce statistically significant improvement from baseline for overall Gram stain skills, where the VUMIE™ did. This may indicate that students did not feel that they had mastered Gram stain skills during the wetlab, because skills are often only able to be completed once due to time and consumables limitations. The virtual simulation, however, allows for deliberate practice (where a learner undertakes a specifically designed activity to improve performance in that given area or skill) and can be used in the domain of mastery learning, where learners participate in an iterative cycle, repeating the learning process until a certain outcome is met [26, 27]. These concepts are particularly applicable to simulations when used to teach or assess a procedure or technique (process-oriented or procedural simulation), like VUMIE™ which teaches the aseptic procedure for various microbiological testing processes [28]. The exact case study can be repeated for all students and will perform in the same manner each time. The results of this study suggest that VUMIE™ could be beneficial as an orientation tool prior to wetlab activities being undertaken, which may improve both the performances and the safety of students during the live laboratory exercises. Similar findings have been reported for other virtual laboratory experiences, particularly for promotion of confidence and more efficient completion of laboratory activities [29, 30].

The delivery of the traditional wetlab allowed for feedback from a demonstrator during the lab session, though students were required to wait until the following week’s session before seeing whether their aseptic technique had been adequate, and their plates had recorded growth. VUMIE™ however, provided instant results and allowed the generation of a lab report where students could see any errors made during the activity. Timely feedback on simulation performance is a critical component of effective learning, encouraging reflective thinking and analysis of learning, so that improvements can be made based on feedback acquired during prior attempts [14, 28].

The ‘anywhere, anytime’ access to virtual learning tools for students has been referred to as ‘simulation on-demand’ and also ‘distributed simulation’, though for the latter term it traditionally referred to a high-fidelity physical unit [28]. Provided the VUMIE™ program is downloaded onto a user’s computer, it can be used anywhere with an internet connection. In addition to the convenience of off-campus use, the VUMIE™ software provided a suitable alternative for several students who were unable to physically take part in the wetlabs. Learning outcomes measurement indicate that there was no significant difference between VUMIE™ and the wetlab, indicating that VUMIE™ could be used again in future where students have contraindications to participating in traditional wetlab activities. In addition to physical safety, the software allows the learner to feel safe in their actions, without fear of negative consequences (such as those that come from making an error in the wetlab). Feeling psychologically safe is associated with better learning outcomes, as students are more likely to treat mistakes as learning opportunities, rather than perceiving them as failures [31, 32].

Not every simulation or virtual laboratory activity will produce successful learning outcomes. Technology-mediated laboratory activities should be used in accordance with preferred instructional design methods and based on sound teaching theories, as well as aligned to curriculum [33, 34]. When virtual activities are used as mere ‘add-ons’ to existing course content, and not directly related to the learning objectives, their usefulness is limited [35, 36]. We have demonstrated that the VUMIE™ software is a useful tool for teaching clinical microbiology to second-year Bachelor of Pharmacy students, however, as is the case with many commercial simulation products, there are components that users might wish to alter. Whilst the program provides an excellent opportunity to practice the interpretation of Gram stains, it does not demonstrate the staining process or the agar plate streaking process. Commercially available products will often deliver many of the requisite educational objectives, however, may not address all of these. If the employer of the simulation is aware of the limitations, learning outcomes can usually still be met using supplementary teaching. Another consideration for future use might be a program that allows modification by the educator or institution.

Another consideration for use of simulations is technology acceptance. For this study, self-reported technology acceptance was overwhelmingly positive. Most respondents were either willing or very willing to use technology ordinarily and reported that they felt having a virtual microbiology training tool available to them would be somewhat or very useful. The technology-acceptance model explains that perceived usefulness and ease of use are predictors of intention to use a simulation or computer-based activity [37]. Incorporating a simulation into a curriculum requires educators to consider the learner and their willingness to use technology, to design a learning activity that will suit the students.

Several factors should be noted when considering future implications and considerations of this research. The VUMIE™ program was accessible by students from the time they attended their first workshop and could be accessed from anywhere provided the student had internet access. Due to privacy reasons there was no way to track how frequently students logged in and used the simulation, including duration of use, or how often simulations were repeated, though this information may assist in understanding and explaining the impact on learning outcomes. Due to ethical guidelines at this institution, surveys must be completely voluntary, which contributed to uneven group numbers (due to attrition). However, a response rate of approximately 50%, which was observed for the post-activity survey compared to the baseline response, is a typical rate of response for data collected from individuals [38]. Furthermore, the ethical restriction on anonymity meant that the participants were responsible for creating and entering their own codes, which may not have been done correctly after the baseline survey was completed. It would have been beneficial to be able to analyse paired data, as well as have the same size group for each survey response set and is something future studies should consider. Despite this limitation the findings of this current study still provide valuable information for other educators. Additionally, this study examined short-term learning outcomes (approximately 8 weeks), whereas long-term retention of learning using the program in comparison to the live wetlab should be investigated for a more rigorous assessment of student learning. Further studies could also examine the integration of the simulation at a chosen time (as is the case with just-in-time simulation), to examine the effects on learning outcomes.

## Conclusion

Our study indicated that the VUMIE™ virtual clinical microbiology simulation program was similarly effective as a traditional wetlab activity in their impact on student learning outcomes. The simulation provided students with a physically and psychologically safe learning environment, with the additional benefits of providing opportunities for students to repeat activities, thus supporting deliberate practice. This suggests that virtual learning tools can, to some extent, replace face-to-face laboratory or clinical teaching or assessment, this being especially useful in a global climate where live teaching is becoming far less frequent.

While the results of this study suggest that a virtual clinical microbiology simulation can produce similar learning outcomes to a traditional wetlab, the research team does not believe that this evidence is sufficient to completely replace the traditional laboratory experience of pharmacy students within their course of study, rather, that it could be considered as a means of training before exposure to a traditional laboratory activity, to enhance deliberate practice for skill acquisition, and as a way of providing a standardised assessment for clinical microbiology education.

## Data Availability

The datasets used and analysed during the current study are available from the corresponding author on reasonable request.

## Abbreviations

GPA: Grade point average
QSEN: Quality and Safety Education for Nurses
